# Acute effects of strength and endurance exercise on serum BDNF and IGF-1 levels in older men

**DOI:** 10.1186/s12877-020-01937-6

**Published:** 2021-01-13

**Authors:** Hamid Arazi, Parvin Babaei, Makan Moghimi, Abbas Asadi

**Affiliations:** 1 Department of Exercise Physiology, Faculty of Sport Sciences, University of Guilan, Rasht, Iran; 2 Cellular and Molecular Research Center, Guilan University of Medical Sciences, Rasht, Iran; 3 Department of Physiology, Guilan University of Medical Sciences, Rasht, Iran; 4 Department of Sport Sciences, Rasht Branch, Islamic Azad University, Rasht, Iran; 5 Department of Physical Education and Sport Sciences, Payame Noor University, Tehran, Iran

**Keywords:** Brain aging, Neurotrophic factor, Physical exercise

## Abstract

**Background:**

Regarding an important effects of physical exercise on brain function in elders, the aim of this study was to examine the effects of strength and endurance exercise on brain neurobiological factors in older men.

**Methods:**

Thirty older men volunteered to participate in this study and were randomly assigned to strength, endurance and control groups. The subjects in strength group performed two circuits of resistance exercise (6 exercises with 10 repetition of 65–70% of one repetition maximum), while endurance group performed 30 min running with 65–70% of maximal heart rate. Blood was obtained pre and post-exercise to determine changes in serum BDNF, IGF-1 and platelets.

**Results:**

After exercise, both the strength and endurance groups showed significant increases in serum BDNF and IGF-1 concentrations and platelets at post-exercise and in comparison to control group (*p* < 0.05). In addition, no statistically significant differences were detected between the strength and endurance groups at post-exercise.

**Conclusion:**

Our findings indicate that both the strength and endurance interventions are effective in elevating BDNF, IGF-1, and platelets, without significant differences between them.

## Background

Biological aging is associated with decreases in fat-free mass (i.e., sarcopenia), muscle strength [1], brain volume size, and decline in cognitive tasks such as memory [2]. In addition, the age-related decreases in muscle mass and strength are by a decline in blood concentrations of circulating anabolic hormones and neurotrophic factors such as BDNF and IGF-1 [3, 4].

BDNF is a protein in the human brain and a member of the neurotrophin family of growth factors which plays an important role in neuronal plasticity and nerve growth during development and adulthood [5, 6]. In addition, IGF-1 as a neurotrophic growth factor influences on nerve growth, neurotransmition, and enhancement of cognitive function [2, 7]. Considering positive association between IGF-1, BDNF and exercise–induced cognitive improvement [5, 8], it has been well reported that these two neurotrophins, more notably BDNF, might be mediators of brain and exercise [5, 9, 10]. In fact, physical exercise enhances growth hormone secretion in the blood circulation which in turn stimulates the production of IGF-1 and then stimulation of brain to enhance of BDNF [5]. Moreover, elevation of platelets post physical exercise is in relation to promotion of blood BDNF because of platelets plays important effects to storage of BDNF. In fact, IGF-1 is a key growth factor which also modulates synaptic plasticity, synapse density, neurotransmission, and even adult neurogenesis and critically involved in vascular maintenance and remodeling, and age-related reductions in IGF-1 have been associated with decreased cerebral vascular density and blood flow [9–14]. Enhanced IGF-1 is also thought to mediate the induction of hippocampal BDNF, and together they are considered as the key factors in the effects of exercise on learning and memory [11].

Knaepen et al. [15] showed that acute endurance but not resistance exercise is effective to increase peripheral BDNF concentrations in healthy individual. However, elevation of BDNF concentrations after endurance exercise has been reported in older women [16], elderly persons with depression, but not on healthy older adults [17].

It has been known that different types of exercise training regulate BDNF, and IGF-1 expression and enhance brain plasticity [4, 12–14]. Despite growing body of literature on acute endurance exercise and BDNF levels [12–17], little is known about the effects of acute resistance exercise on this issue. Some studies exploring the effects of acute resistance exercise on BDNF levels failed to find a significant response [18–20] due to variety in intensity and duration of physical activity. For example, Swift et al. [18] used low-to-moderate intensities with fruitless results. Moreover, considering the fact that resistance exercise leads to elevation of platelets, it should be noted that platelets plays important area to storage of BDNF, interfere on serum BDNF level [11]. To our knowledge, there is no evidence comparing the influence of acute resistance and endurance exercise on serum level of IGF-1, BDNF, and platelets in older adults. Therefore, the aim of this study was to examine the effects of strength and endurance exercise on serum BDNF, IGF-1 and platelets and determine the association between these variables in older men.

## Methods

### Participants

Initially, 40 older men volunteered to participate in the study. They were recruited through advertisement and letters from province basketball board. The inclusion criteria were: 1) at least 10 years of experience in basketball, 2) being healthy by self-report (i.e., completion of the revised physical activity readiness questionnaire for adults older), 3) lack of cardiovascular system and musculoskeletal diseases which checked by physician, 4) not using any drugs and supplements. Before initiating the experiment, all subjects were screened by a physician using an extensive medical history, and finally 30 subjects were selected for this study. After anthropometric measurements, the subjects were matched based on age and body weight and then were randomly assigned to one of the three groups: strength group (*n* = 10, age = 60.8 ± 1.8 y, height = 177.8 ± 8.9 cm, weight = 90.6 ± 16 kg, and body fat = 21.9 ± 3.7%), endurance group (*n* = 10, age = 60.7 ± 1.7 y, height = 181 ± 5.4 cm, weight = 85.9 ± 13.4 kg, and body fat = 21.8 ± 2.8%), and control group (*n* = 10, age = 60.9 ± 0.9 y, height = 179.6 ± 8.2 cm, weight = 87.7 ± 9.7 kg, and body fat = 21.0 ± 3.9%) using computer-generated random numbers. Before participating to in any measurements, the participants were informed about the procedures of the study, benefits and possible risks associated with the study and signed a written consent form which was approved by the Declaration of Helsinki and Research Committee at the Islamic Azad University.

### Study design

This study utilized a controlled, randomized design. Subjects in strength and endurance exercise groups were recruited to the laboratory on three and two occasions with 48 h apart at 9–11 AM, respectively. On the first visit, subjects were familiarized with exercise and testing procedures. Subjects’ characteristics such as; age, height, weight, and percent body fat via 3-site skinfolds were measured at familiarization session. Subjects in strength group also performed 1 repetition maximum (1RM) test for the leg press, arm curl and calf rise. On the second visit, 1RM of bench press, knee flexion and lat pull down were determined for only strength group. On the third visit, subjects in the strength group completed two circuits of resistance exercise, which consisted of 10 repetitions with 65–70% of 1RM. Subjects in the endurance group, also performed 30-min (3 × 10-min) endurance exercise with 65–70% of maximal heart rate (MHR). Blood samples were also collected to analyze brain neurutrophins at pre and post exercise. Subjects were instructed to maintain their normal caloric intake throughout the duration of the study. The subjects in the control group did not perform any exercise and were in the training room and laboratory to do measurements similar to treatment groups.

#### Anthropometric measurements

Height was measured using a wall mounted stadiometer to the nearest 0.5 cm (Seca 222, Terre Haute, IN). Weight was measured using a digital scale to the nearest 0.1 kg (Tanita, BC-418MA, Tokyo, Japan). Percent body fat was assessed using skinfolds with Lafayette caliper (Skin Fold Caliper, Model 01127 INDIANA), following a previously described [21]. A 3-site (chest, abdominal, and thigh) skinfold equation was used to obtain percent body fat using Jackson and Pollock equation [22].

#### 1RM testing

For the prescription of resistance exercise, 1RM strength was assessed in the free-weight (i.e. arm curl, and bench press) or machine exercises (i.e. leg press, calf rise, knee flexion, and lat pull down) using previously described methods [23]. Subjects performed a warm-up specific to this test consisting of eight to ten repetitions using a light weight (i.e., 50% of 1RM), three to five repetitions using a moderate weight (i.e., 75% of 1RM), and one to three repetitions using a heavy weight (i.e., 90% of 1RM). Then, resistance was increased until participants were unable to complete an attempt using proper technique through a full range of motion. Up to five maximal attempts were allowed, with 5 min of rest between attempts. Spotters and investigators were present to provide verbal encouragement and ensure safety.

#### Exercise interventions

Each exercise intervention lasted 45 min (i.e., 10 min of standard warm up, 30 min of main exercise, and 5 min of cool down). The participants received verbal encouragement throughout the exercise session. An experienced strength and conditioning coach monitored all exercise protocols.

#### Strength exercise

Subjects in strength group performed circuit type resistance exercise (two circuits) in order to leg press, lat pull down, knee flexion, bench press, calf rise and arm curl for 10 repetitions with 65–70% of 1RM. The rest period between stations and circuits were 60 and 120 s, respectively.

#### Endurance exercise

To main exercise for the endurance group consisted of 30 min (3 × 10-min with 120 s interval) of running with 65–70% of MHR (208–0.7 (age); approximately 100-to-110 beat/min). The MHR was assessed using Polar S610i heart rate monitor (FIN, 90440, FINLAND).

#### Blood sampling and analysis

Blood samples were obtained (10 cc) from the antecubital vein at pre, and post exercise at sitting position (i.e., 10 min) to control hemoconcentration in alteration of BDNF and IGF-1 levels. Pre blood samples were drawn following a 10 min equilibration period prior to exercise. Post blood samples were taken within 5 min of exercise cessation reported by Rojas Vega et al. [24] to determine serum BDNF levels at post-exercise. The blood samples were assigned to clot at room temperature for 30-min and centrifuged at 3000×*g* for 15-min. The serum layer was removed and frozen at − 80 °C for further analyses. The blood samples of participants were measured in duplicate and were decoded only after completing the analyses. Serum IGF-1 was measured by commercially available enzyme-linked immunosorbent assay (ELISA) kit (Diamerta, R & D manufacturing, ITALY) according to the manufacturer’s procedures. The sensitivity of the IGF-1 assay was 0.13 ng/mL. The intraassay coefficient of variation (CV) for IGF-1 was less than 6%. Serum BDNF was assayed in duplicate according to the manufacturer’s instructions (R&D systems, Inc. Minneaplis, USA). The sensitivity of the BDNF assay was 0.13 ng/mL. The intraassay CV for BDNF was less than 5%. EDTA-treated whole blood was used for platelets assessment via automated clinical analyzer (Quest Diagnostics Inc. Willimantic, CT).

### Statistical analyses

All values are presented as the mean ± standard deviation (SD). Relative changes (%) in the dependent variables and effect sizes (ESs) are expressed as 95% confidence limits (CL). Normality of all data before and after interventions was checked with the Shapiro–Wilk test. To determine the effect of interventions on neurobiological changes a 2-way variance analysis with repeated measurements (3 [groups] × 2 [times]) was applied. When a significant F value was achieved, Bonferroni post-hoc procedures were performed to identify the pairwise differences between the means, and one-way ANOVA’s were used to compare changes among the groups (strength, endurance and control). Pearson product moment correlations coefficient (r) was used to determine relationship between BDNF, IGF-1, and platelets using post-test scores. The level of significance was set at *p* ≤ 0.05. Threshold values for assessing magnitudes of ES were < 0.2, trivial; 0.2–0.6, small; 0.6–1.2, moderate; 1.2–2.0, large; 2.0–4.0, very large; and > 4.0, nearly perfect [25].

## Results

The subjects in the control group did not show any significant changes in the variables and experimental groups indicated significant differences compared to control groups in all variables (*p* ≤ 0.05).

**Fig. 1:**
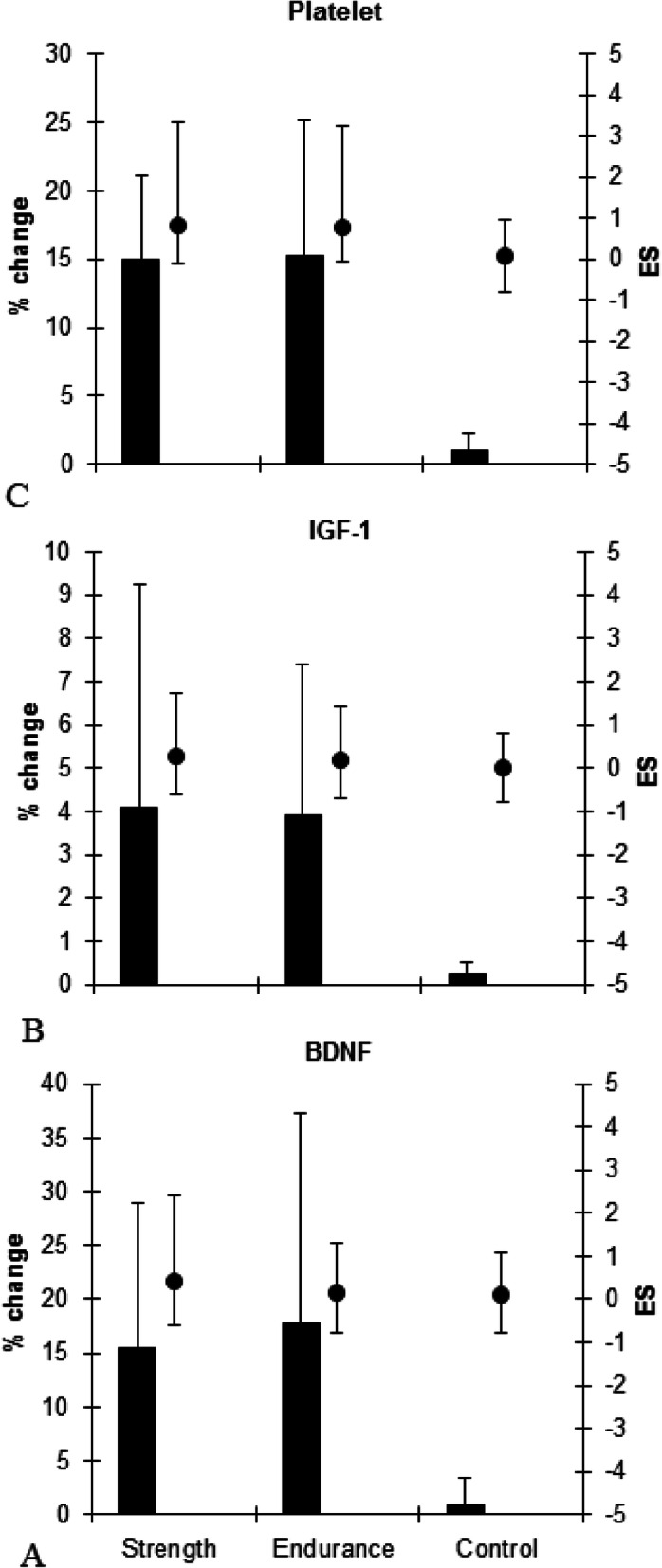
Percent change and effect size (95% CI) in the variables (BDNF (**a**), IGF-1 (**b**) and Platelet (**c**)) for the groups. ES: Effect size. BDNF: Brain derived neurotrophic factor. IGF-1: Insulin-like growth factor 1

The endurance and strength groups demonstrated small to moderate significant increases (*p* = 0.003, F = 15.642) in the BDNF levels from pre to post-exercise (endurance, ES = 0.15 (− 0.7, 1.02 CI, small), 17.7%, *p* = 0.002; strength, ES = 0.42 (− 0.48, 1.29 CI, moderate), 15.5%, *p* = 0.003). After exercise intervention, no significant differences were observed between the endurance and strength groups in the BDNF levels (*p* ≥ 0.05) (Table 1 and Fig. 1a).

**Table 1:** Changes in the variables from before to after exercise among the groups

	Strength (***n*** = 10)	Endurance (***n*** = 10)	Control (***n*** = 10)	Significant
**BDNF (ng/mL)**				
Pre	3.58 ± 1.05	4.13 ± 3.65	3.18 ± 0.27	G = 0.413
Post	4.16 ± 1.82 *, **	4.68 ± 3.9 *, **	3.21 ± 0.29	T = 0.003
				G × T = 0.023
**IGF-1 (ng/mL)**				
Pre	134.4 ± 21.6	157.1 ± 30.84	121.7 ± 17.7	G = 0.13
Post	139.5 ± 20 *, **	162.6 ± 28.86 *, **	121.9 ± 17.6	T = 0.03
				G × T = 0.039
**Platelet (micl× 1000)**				
Pre	246.9 ± 41.1	244.8 ± 46.6	217.6 ± 34.2	G = 0.17
Post	283.6 ± 47.7 *, **	279.4 ± 40.5 *, **	219.7 ± 34	T = 0.001
				G × T = 0.001

* Denotes significant difference from corresponding before exercise (*p* ≤ 0.05). ** Denotes significant difference from corresponding control group (*p* ≤ 0.05). Values are mean ± SD. *BDNF:* Brain derived neurotrophic factor. *IGF-1:* Insulin-like growth factor 1

The endurance and strength groups demonstrated small to moderate significant increases (*p* = 0.03, F = 16.013) in the IGF-1 levels from pre to post-exercise (endurance, ES = 0.18 (− 0.7, 1.05 CI, small), 3.91%, *p* = 0.02; strength, ES = 0.29 (− 0.6, 1.16 CI, moderate), 4.1%, *p* = 0.03). In addition, there were no differences at post exercise between the endurance and strength groups in IGF-1 level (*p* ≥ 0.05); however, strength group indicated more ES (moderate vs. small) than endurance group (Table 1 and Fig. 1b).

In platelets, both the endurance groups indicated moderate significant (*p* = 0.001, F = 104.989) improvements from pre to post-exercise (ES = 0.79 (− 0.15, 1.67 CI, moderate), 15.2%, *p* = 0.001) and strength (ES = 0.82 (− 0.12, 1.70 CI, moderate), 14.9%, *p* = 0.001). After exercise intervention, no significant differences were observed between the endurance and strength groups in the platelets (*p* ≥ 0.05) (Table 1 and Fig. 1c).

**Fig. 2:**
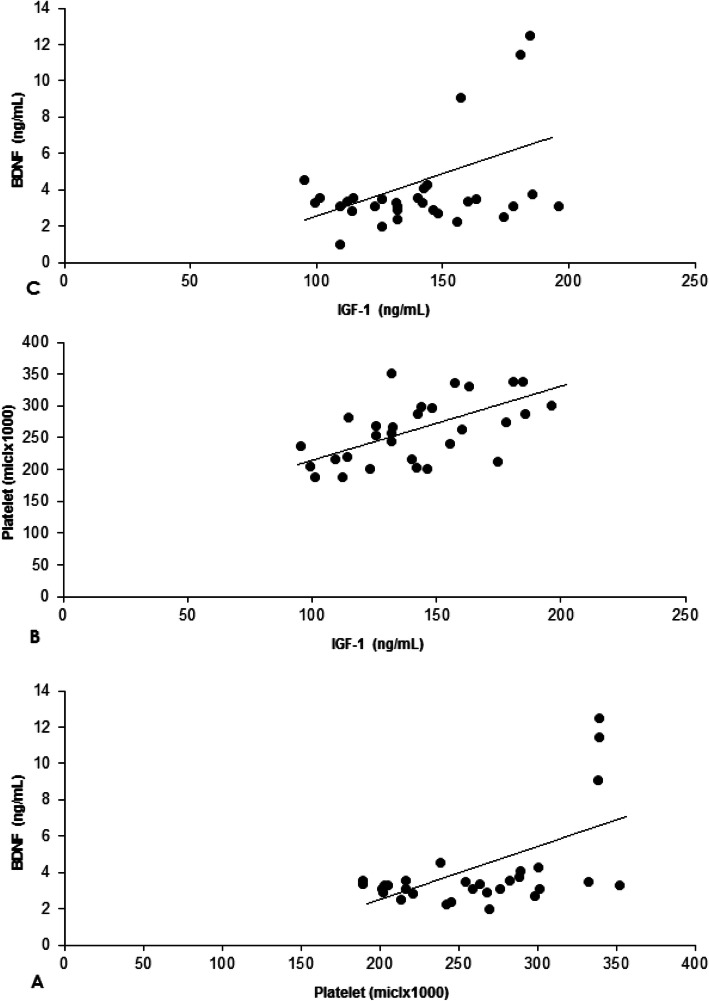
Correleations between dependent variables (Platelet and BDNF (**a**), IGF-1 and Platelet (**b**), IGF-1 and BDNF (**c**)) at post-exercise value for all subjects (*n* = 30). BDNF: Brain derived neurotrophic factor. IGF-1: Insulin-like growth factor 1

Statistically significant correlations were detected between IGF-1 level with platelets (*r* = 0.556, *p* = 0.001) and BDNF (*r* = 0.347, *p* = 0.042) (Fig. 2b and c). Also, positive correlations were observed between serum BDNF level and platelets (r = 0.535, *p* = 0.002) (Fig. 2a).

## Discussion

Our findings revealed that both the acute strength and endurance exercise induced meaningful changes in BDNF, IGF-1 and platelets in older adults and there were no statistically significant changes between the treatment groups in the variables at post-exercise. We also found significant positive correlations between IGF-1, BDNF and platelets following the exercise intervention. In line with our findings, previous studies [5, 6, 18, 20] reported that endurance and resistance exercise has positive effects for elevating of BDNF levels in older subjects. However, Rojas Vega et al. (2008) reported no significant changes in serum BDNF in response to endurance exercise [26]. The discrepancy in the magnitude of changes in BDNF following the exercise intervention could be due to the intensity of exercise which plays an importance role in stimulating secretion of BDNF. Similar to previous studies [24–26], peripheral measures of circulating BDNF was measured to identify the effects of exercise. It appears that peripheral serum BDNF level has the efficacy in showing the level of BDNF in the brain [27]. In fact, it is known that the peripherally produced BDNF can exert trophic effects to support the CNS, because it can cross the blood—brain barrier. At rest, the production of BDNF is regulated by neuronal activity; however, physiological stimulation such as physical exercise can be used to regulate of the BDNF levels in the brain [27]. The regulation of BDNF during physical exercise could open up an interesting perspective for the function of BDNF in neuroplasticity of the hippocampus, cerebellum and frontal cortex [28]. Thus, the increase of the serum peripheral BDNF level following the physical exercise might be of importance in maintaining a proper brain function regarding positive correlations between concentrations of central (neural) and peripheral measures of BDNF [29, 30].

Following strength and/or endurance exercise mitogen-activated protein kinas (an important intracellular signal pathway involved in BDNF production and secretion) stimulation is increased in hippocampus and brain cortex [29] resulting in serum BDNF level enhancements. In fact, performing exercise may induces maturation of BDNF (i.e., mBDNF) by stimulation of Ca2+/calmodulin-dependent protein kinase II (CaMKII) and Synapsin I, and also increases in expression and activation of tissue plasminogen activator (tPA) and therefore enhancement of proteolytic mRNA of mBDNF [30]. However, in this study we did not directly measure molecular pathway of BDNF secretion in the circulation. Collectively, increases in IGF-1 levels could be a key explanation to increases in BDNF levels following exercise because of IGF-1 could plays an important role for the transformation of proBDNF to mBDNF in CNS resulting in enhancements of serum BDNF [24, 31]. In addition, increases in platelets at post exercise could be another important mechanism to increase BDNF levels because of Reddy Kilim and Lakshmi [32] reported that platelets increased immediately post exercise due to release of fresh platelets from the bone marrow and maintained the elevation of blood neurotrophins (i.e., BDNF) for 5 min after exercise and then declined to baseline levels following (i.e., 10 min) physical exercise [24]. However, more studies are necessary to clarify the effects of exercise on peripheral BDNF and their effects on brain BDNF levels in humans.

Following strength and endurance exercise, serum IGF-1 increased significantly compared with pre-exercise. However, the magnitude of changes in IGF-1 secretion was greater for the strength group compared to endurance group (ES; 0.29 [moderate] vs. 0.18 [small]). These findings are in accordance with previous studies which reported the importance of resistance exercise to increase IGF-1 concentrations in elders [1–3, 5]. The elevation in IGF-1 after exercise could be due to increases in growth hormone (GH). In fact, blood lactate concentration increased after exercise and stimulates hypothalamus to release of GH in the blood and consequently induces IGF-1 secretion [1, 3]. Considering positive effects of IGF-1 on BDNF levels [33], IGF-1 could be a factor contributing to neural cell growth, differentiation and survival.

Both the strength and endurance exercises induced statistically significant increases in platelets after exercise. These findings are in line with previous investigations which used resistance and endurance exercise [34, 35]. Increases in circulating platelets after exercise might be related to elevation in epinephrine which facilitates splenic release of platelets [34–36]. Another potential mechanism to explain exercise-induced increase in platelets could be increases in shear stress following both the endurance and strength exercise [31, 35]. In fact, strenuous physical exercise induced meaningful increases in both platelet count and activation induced by increased plasma platelet factor 4 and β-thromboglobulin [32]. According to the results of the present study both the strength and endurance exercise groups induced similar responsibility to produce stimulations in platelets and also similar increases in BDNF signaling pathways and IGF-1 secretion. Taken together, physical exercise (i.e., strength and/or endurance) not only increased IGF-1 secretion but also increased platelets and serum BDNF level in older adults. This finding theoretically confirms the high capacitance of brain neurotrophic factors even in old ages, to boost neuronal plasticity, neurogenesis and cognitive functions [37, 38].

## Conclusion

The present study suggests that a session of physical exercise (i.e., strength or endurance) have meaningful effects to enhance IGF-1, platelets and BDNF levels in older men. Therefore, it could be recommend that older adults use these exercise protocols as no pharmacological tool to increase serum IGF-1 and BDNF levels and possibly enhance brain function in previously active men (i.e., basketball players); however, considering ES and % change in the variables the strength and endurance exercise group indicated no significant differences between their changes and both interventions induced similar effects.

## Data Availability

The datasets during and/or analysed during the current study available from the corresponding author on reasonable request.

## Abbreviations

IGF-1: Insulin-like growth factor 1
BDNF: Brain-derived neurotrophic factor
1RM: 1 Repetition maximum
MHR: Maximal heart rate
ELISA: Enzyme-linked immunosorbent assay
CV: Coefficient of variation
ES: Effect size
CL: Confidence limits
CaMKII: Ca^2+^/calmodulin-dependent protein kinase II
tPA: Tissue plasminogen activator
mBDNF: Mature BDNF
GH: Growth hormone

## References

[1] Kraemer WJ, Hakkinen K, Newton RU, Nindl BC, Volek JS, Mccormick M (1999). Effects of heavy-resistance training on hormonal response patterns in younger vs. older men. J Appl Physiol.

[2] Doi T, Shimada H, Makizako H, Tsutsumimoto K, Hotta R, Nakakubo S, Suzuki T (2015). Association of insulin-like growth factor-1 with mild cognitive impairment and slow gait speed. Neurobiol Aging.

[3] Hakkinen K, Pakarinen A, Kraemer WJ, Hakkinen R, Valkeninen H, Alen M (2001). Selective muscle hypertrophy, changes in EMG, and force, and serum hormones during strength training in older women. J Appl Physiol.

[4] Etnier JL, Wideman L, Labban JD, Piepmeier AT, Pendleton DM, Dvorak KK, Becofsky K (2016). The effects of acute exercise on memory and brain-derived Neurotrophic factor (BDNF). J Sport Exerc Psychol.

[5] Maass A, Düzel S, Brigadski T, Goerke M, Becke A, Sobieray U (2016). Relationships of peripheral IGF-1, VEGF and BDNF levels to exercise-related changes in memory, hippocampal perfusion and volumes in older adults. NeuroImage.

[6] Tsai SW, Chan YC, Liang F, Hsu CY, Lee IT (2015). Brain-derived neurotrophic factor correlated with muscle strength in subjects undergoing stationary bicycle exercise training. J Diabetes Complicat.

[7] Ashpole NM, Sanders JE, Hodges EL, Yan H, Sonntag WE (2015). Growth hormone, insulin-like growth factore-1 and the aging brain. Exp Gerontol.

[8] Ding Q, Vaynman S, Akhavan M, Ying Z, Gomez-Pinilla. Insulin-like grouth factor 1 interfaces with brain-derived neurotrophic factor-mediated synaptic plasticity to modulate aspects of exercise-induced cognitive function. Neuroscience 2006;140:823–833. 10.1016/j.neuroscience.2006.02.084 16650607

[9] Trejo JL, Piriz J, Llorens-Martin MV, Fernandez AM, Bolos M, LeRoith D, Nunez T-AI (2007). Central actions of liver-derived insulin-like growth factor I underlying its pro cognitive effects. Mol Psychiatry.

[10] Bibel M, Barde YA (2000). Neurotrophins: key regulators of cell fate and cell shape in the vertebrate nervous system. Genes Dev.

[11] Fujimura H, Altar CA, Chen R, Nakamura T, Nakahashi T, Kambayashi J (2002). Brain derived neurotrophic factor is stored in human platelets and released by agonist stimulation. Thromb Haemost.

[12] Hillman CH, Snook EM, Jerome GJ (2003). Acute cardiovascular exercise and executive control function. Int J Psychophysiol.

[13] Pontifex MB, Hillman CH, Fernhall B, Thompson KM, Valentin TA (2009). The effect of acute aerobic and resistance exercise on working memory. Med Sci Sports Exerc.

[14] Tomporowski PD, Lambourne K, Okumura MS (2011). Physical activity interventions and children’s mental function: an introduction and overview. Prev Med.

[15] Knaepen K, Goekint M, Heyman EM, Meeusen R (2010). Neuroplasticity — exercise induced response of peripheral brain-derived neurotrophic factor: a systematic review of experimental studies in human subjects. Sports Med.

[16] Gomes WF, Lacerda AC, Mendonca VA, Arrieiro AN, Fonseca SF, Amorim M, Teixeira AL, Teixeira MM, Miranda AS, Coimbra CC, Brito-Melo GE (2013). Effect of exercise on the plasma BDNF levels in elderly women with knee osteoarthritis. Rheumatol Int.

[17] Laske C, Banschbach S, Stransky E, Bosch S, Straten G, Machann J, Fritsche A, Hipp A, Niess A, Eschweiler GW (2010). Exercise-induced normalization of decreased BDNF serum concentration in elderly women with remitted major depression. Int J Neuropsychopharmacol.

[18] Swift DL, Johannsen NM, Myers VH, Earnest CP, Smits JA, Blair SN, Church TS (2012). The effect of exercise training modality on serum brain derived neurotrophic factor levels in individuals with type 2 diabetes. PLoS One.

[19] Goekint M, De Pauw K, Roelands B, Njemini R, Bautmans I, Mets T, Meeusen R (2010). Strength training does not influence serum brain-derived neurotrophic factor. Eur J Appl Physiol.

[20] Forti LN, Njemini R, Beyer I, Eelbode E, Meeusen R, Mets T, Bautmans I (2014). Strength training reduces circulating interleukin-6 but not brain-derived neurotrophic factor in community-dwelling elderly individuals. Age (Dordr.).

[21] Arazi H, Damirchi A, Asadi A (2013). Age-related muscle circumference, strength development and hormonal adaptations with 8 weeks moderate intensity resistance training. Ann Endocrinol.

[22] Jackson AS, Pollock ML (1985). Practical assessment of body composition. Phys Sports Med.

[23] Kraemer WJ, Fry A (1995). ACSM’s guidelines for exercise testing and prescription.

[24] Rojas Vega S, Struder HK, Vera WB (2006). Acute BDNF and cortisol response to low intensity exercise and following ramp incremental exercise to exhaustion in humans. Brain Res.

[25] Hopkins WG, Marshall SW, Batterham AM, Hanin J (2009). Progressive statistics for studies in sports medicine and exercise science. Med Sci Sports Exerc.

[26] Rojas Vega S, Abel T, Lindschulten R (2008). Impact of exercise on neuroplasticity-related proteins in spinal cord injured humans. Neuroscience.

[27] Piepmeier AT, Etiner JL (2015). Brain-derived neurotrophic factor (BDNF) as a potential mechanism of the effects of acute exercise on cognitive performance. J Sport Health Sci.

[28] Vaynman S, Gomez-Pinilla F (2005). License to run: exercise impacts functional plasticity in the intact and injured central nervous system by using neurotrophins. Neurorehabil Neural Repair.

[29] Zoladz JA, Pilc A, Majerczak J (2008). Endurance training increases plasma brain-derived neurotrophic factor concentration in young healthy men. J Physiol Pharmacol.

[30] Sartori CR, Vieira AS, Ferrari EM (2011). The antidepressive effect of the physical exercise correlates with increased levels of mature BDNF, and proBDNF proteolytic cleavagerelated genes, p11 and tPA. Neuroscience.

[31] Mejri S, Bchir F, Ben Rayana MC (2005). Effect of training on GH and IGF-1 responses to a submaximal exercise in football players. Eur J Appl Physiol.

[32] Reddy Kilim S, Lakshmi PV (2015). A study on effect of severity of exercise on platelet function. J Evolution Med Dental Sci.

[33] Lee KJ, Rhyu IJ (2009). Effects of exercise on structural and functional changes in the aging brain. J Korean Med Assoc.

[34] Ahmadizad S, El-Sayed MS (2003). The effects of graded resistance exercise on platelet aggregation and activation. Med Sci Sports Exerc.

[35] Ahmadizad S, El-Sayed MS, Maclaren DP (2006). Responses of platelet activation and function to a single bout of resistance exercise and recovery. Clin Hemorheol Microcirc.

[36] Peat EE, Dawson M, McKenzie A, Hillis WS (2010). The effects of acute dynamic exercise on haemostasis in first class Scottish football referees. Br J Sports Med.

[37] Goekint M, Roelandsa B, Pauw KD, Knaepen K, Bosa I, Meeusen R (2010). Does a period of detraining cause a decrease in serum brain-derived neurotrophic factor?. Neurosci Lett.

[38] Borror A (2017). Brain-derived neurotrophic factor mediates cognitive improvements following acute exercise. Med Hypotheses.

